# Analysis of Single Nucleotide Polymorphisms within ADAM12 and Risk of Knee Osteoarthritis in a Chinese Han Population

**DOI:** 10.1155/2015/518643

**Published:** 2015-01-15

**Authors:** Lin Wang, Lin Guo, Fengde Tian, Ruihu Hao, Tiejun Yang

**Affiliations:** Department of Orthopaedics, The Affiliated Zhongshan Hospital of Dalian University, No. 6 Jiefang Road, Da Lian, China

## Abstract

*Objective*. Osteoarthritis (OA) is a complex arthritic condition in which the genetic factor plays a major role. One of the candidate genes of is the ADAM12 gene, but no consistency has been reached till now. This study aims to investigate the potential role of four single nucleotide polymorphisms (SNPs) of the ADAM12 gene in susceptibility to knee OA and its progression in Chinese Han population. *Methods*. The rs1278279, rs3740199, rs1044122, and rs1871054 polymorphisms were genotyped and compared in a population based cohort consisting of 164 OA subjects and 200 age- and gender-matched controls. *Results*. The SNP rs1871054 was found with increased risk of OA susceptibility in comparing the genotype frequencies between the case and control groups no matter for which model of comparison (allele level, dominant model, recessive model, and extreme genotype model). Additionally, the SNP rs1871054 was found associated with increased OA severity according to the K/L grade. *Conclusion*. In summary, we have identified that the rs1871054 variant within the ADAM12 gene is a risk factor for increased osteoarthritis susceptibility and severity.

## 1. Introduction

Osteoarthritis (OA) is a complex arthritic condition, evolving over decades and leading to the loss of joint function. Prevalence of OA increases with age: the disease affects 10% of males and 18% of females over 45, and these figures are predicted to rise as the general population ages. It is expected that, by 2030, 20% of adults in Western Europe and North America will have OA. It is characterized by the degradation of articular cartilage, the modification of the subchondral bone, and the inflammation of the synovial membrane [1]. OA causes joint pain, stiffness, and loss of function, predominantly affecting the knee, hip, hand, spine, and other weight-bearing joints. The main recommendations for OA management concentrate on the control of symptoms, that is, pain and function [2–6]. This goal is supposed to be achieved mostly by the use of acetaminophen or nonsteroidal anti-inflammatory drugs (NSAIDs). So far, none of the available treatment allows the control or, even better, the arrest of the disease progression. OA is primarily associated with aging; however, there are other important contributing factors, including obesity, history of joint trauma, repetitive joint use, genetics, inherited and acquired metabolic disorders, muscle weakness, underlying anatomical and orthopedic disorders (e.g., congenital hip dislocation), joint infection, crystal deposition, previous rheumatoid arthritis, and a variety of bone turnover and blood clotting disorders. Nowadays, the individual susceptibility difference was mostly related to the genetic factors. To date, several genomewide linkage analyses (GWAS) and numerous association studies of candidate genes have been performed to disclose genetic pattern of OA. Until now, promising but contradictory data are published for the association of a member of disintegrin and metalloproteinase family—ADAM12 with the pathogenesis of OA [7–9]. ADAM12 is an active proteinase, which is highly expressed in remodelling and fast-growing tissues such as the placenta and malignant tumours. One of the splice variant of ADAM12 was found to be overexpressed in human OA serum and cartilage [10, 11]. Additionally, the single nucleotide polymorphism (SNP) rs1871054 in ADAM12 gene was recognized as a risk predictor in multiplicative knee OA model [12]. However, contradictory data exist recognizing the SNPs in ADAM12 with no relationship with OA susceptibility [8, 13]. The inability to replicate the association between the ADAM12 SNPs and OA susceptibility limits the application in clinical use and the research of the molecular mechanism. This study aims to investigate the association between ADAM12 SNPs and knee OA susceptibility in Chinese Han population.

## 2. Method

### 2.1. Study Population

A total of 164 patients diagnosed with primary knee OA and 200 age-matched unrelated healthy controls were recruited. All patients and controls recruited in this study were Chinese Han population. The Han ethnic group makes up 92% of the population in China and 20% of the global population, making it the largest ethnic group in the world. This study was performed from 2011 to 2014 and carried out in accordance with the Helsinki Declaration II and European Guidelines for Good Clinical Practice. It was approved by the ethics committee of the affiliated Zhongshan Hospital of Dalian University and written informed consent was obtained from all the patients and control participants. The diagnosis of knee OA was based on the criteria of the American College of Rheumatology, which included primary OA with any symptoms of one or two knees and radiographic signs of OA according to the Kellgren-Lawrence (K/L) grading system (≥2 scale). Other etiologies of knee joint disease such as posttraumatic or postseptic arthritis, inflammatory arthritis, and skeletal or developmental dysplasia were excluded from the study. Healthy control subjects had no symptoms or signs of joint disease including pain, swelling, tenderness, or restriction of movement, and standard X-rays of the knee joints confirmed no signs of OA.

### 2.2. Genotyping

DNA was extracted from 2 mL venous blood using a QIAGEN kit according to the manufacturer's instructions (QIAGEN, Hilden, Germany) and stored at −20°C before genotyping. The alleles of SNPs in the ADAM12 gene were detected by using the improved multiplex ligase detection reaction (iMLDR) method (Shanghai Genesky Bio-Tech Co., Ltd.; http://www.geneskies.com/). Genotyping accuracy in the samples was confirmed by direct sequencing of PCR products for 5% randomly chosen samples. Genotypes were determined by independent investigators who were blinded to patients' identities and phenotypes.

### 2.3. Statistical Analysis

The demographic and clinical data were presented as Mean ± SD and compared between groups by Student's *t*-tests in this study. The Statistical Package for Social Sciences software (SPSS, Inc., Chicago, IL, USA), version 18.0 for Windows, and the HaploView software were used for statistical analysis in this study. The association between the ADAM12 polymorphisms and OA susceptibility was assessed under the following genetic models, which were treated as a dichotomous variable: (i) m-allele (minor) versus M-allele (major) for allele level comparison; (ii) mM + MM versus mm for a dominant model of the M-allele; (iii) MM versus mM + mm for a recessive model of the M-allele; and (iv) MM versus mm for the extreme genotype. Multivariate logistic regression was used to estimate odds ratios (ORs) and 95% confidence intervals (CI) after adjustment for age, gender, and BMI. The linkage disequilibrium (LD) mapping and the associations between haplotypes of selected SNPs and risk of OA were estimated by the HaploView software. The *P* < 0.05 was considered to indicate a statistically significant difference.

## 3. Results

### 3.1. Characteristics of Study Subjects

Demographic data of the population studied and the number of individuals in each group are shown in Table 1. There were no significant differences between groups in terms of age, gender, and mean body mass index (BMI). In the knee OA patients, the mean age was 67.4 ± 4.2 years. In the healthy controls, the mean age was 65.9 ± 5.3 years (n.s). The male/female ratio was 58/106 in the knee OA patients and 62/138 in the controls (n.s). Furthermore, the mean BMI value was not significantly different between groups, 25.3 ± 4.2 in the knee OA patients and 24.4 ± 4.5 kg/m^2^ in the controls, respectively (n.s).

### 3.2. Association of ADAM12 Polymorphisms with OA Susceptibility

As expected, the distribution of the genotype of SNP rs1278279, rs3740199, rs1044122, and rs1871054 conformed to the Hardy-Weinberg equilibrium and the genotyping success rate was 100%.  Table 2 listed the genotyped and allele distributions of the SNPs for the cases and controls. The SNP rs3740199, rs1044122, and rs1278279 were found with no statistical difference in comparing the genotype frequencies between the case and control groups no matter for which model of comparison (allele level, dominant model, recessive model, and extreme genotype model). However, for the SNP rs1871054, the C allele was associated with an increased risk of OA in terms of the frequency of allele comparison (C versus T, OR = 1.84; 95% CI = 1.57 to 2.23, *P* < 0.0001). For a dominant model of the C allele, the CT + CC genotypes were associated with the risk for OA (CT + CC versus TT, OR = 1.64, 95% CI = 1.01 to 2.74, *P* = 0.0348). For a recessive model of the C allele, the CC homozygote genotype was also associated with increased susceptibility to OA (CC versus CT + TT, OR = 2.65, 95% CI = 1.72 to 4.14, *P* < 0.0001). For the extreme genotype, the CC genotypes were associated with the risk for OA (CC versus TT, OR = 2.78, 95% CI = 1.56 to 4.95, *P* = 0.0012).

**Figure 1:**
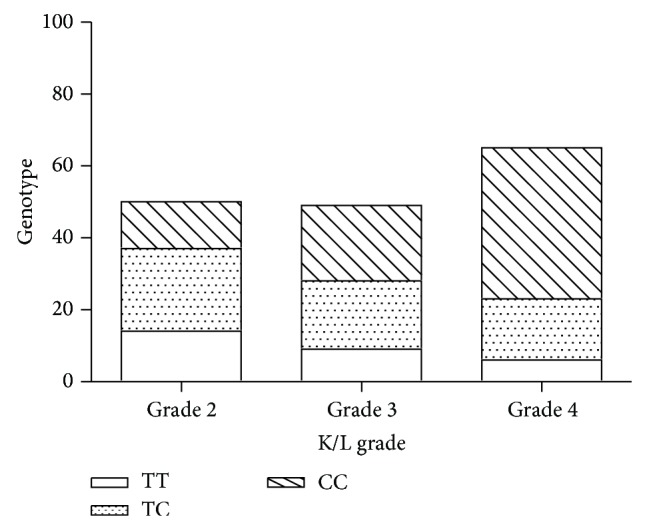
The analysis of the genotype frequency of ADAM12 SNP rs1871054 with different KL grade in the cases.

Also, the genotype frequency of ADAM12 SNP rs1871054 was analyzed according to the KL grade in the cases. The severity of OA was found significantly associated with the C allele frequency (*P* = 0.0011), as in Figure 1.

## 4. Discussion

In the present study, we evaluated the relation of the ADAM12 SNPs to knee OA susceptibility and severity. Our study demonstrated that the ADAM12 polymorphism rs1871054 was associated with increased risk and severity of OA. The genetic background is essential determinants of OA. Extensive functional genomic research (DNA and RNA) on relevant joint tissue, cell, and animal models is needed to discover novel unknown members and elucidate mechanisms of current OA susceptibility genes and pathways [14]. Identification of OA susceptibility genes could make it possible in the future to predict disease phenotypes as well to construct OA prediction models based on genotype information [15]. Polymorphisms of ADAM12 gene were reported to be associated with knee OA development and progression [7, 10, 16, 17]. Nevertheless, these results are not consistent as some other studies failed to replicate this association in other populations or races [8, 13]. Lack of replication makes it difficult for the application of clinical use. The reasons could be due to genotyping different and too few markers; differences in case ascertainment and phenotype criteria; differences in ethnicity; and the occurrence of false negatives in the replication study or false positives in the initial studies. Insufficient power related to sample size is a likely source of false positives in initial studies, which consequently tend to overestimate genetic effects “winners curse.” Limited power to detect genetic associations is a significant problem in studying genetics of any complex disease. This study is based on a relatively small population, but the results make it possible to perform a meta-analysis to avoid the limited study power.

The matrix metalloproteinases, A Disintegrin and Metalloproteinase (ADAMs), are the main proteolytic enzymes that regulate extracellular matrix turnover in the cartilage [18]. The ADAM12 is a catalytically active metalloproteinase, which is expressed mainly in remodeling and fast-growing tissues. ADAM12 is a multifunctional zinc-dependent protease with the ability to shed membrane-anchored proteins like cytokines, growth factors, and their receptors. It has been proposed that cleavage of these substrates may regulate availability of bioactive molecules and thereby also regulate inflammation, tissue vascularization, or remodeling [19]. Indeed, according to published data, ADAM12 appears to modulate mesenchymal cell differentiation and could therefore be involved in remodeling of different tissues. The ADAM12 has been shown to be involved in growth factor shedding [20], cell adhesion, and fusion [21, 22], which suggest also its role in inflammatory and immune reactions. Therefore, ADAM12 has been shown to be upregulated in different cancer types, pregnancy disorders, and human OA cartilage [7, 10, 23].

The reason for the discrepancy regarding the association of the SNPs with OA between the present study and the previous studies is unclear, but several possibilities are conceivable. The discrepancy could be attributed to the different characteristics of the cohorts, such as age, gender, population, or sample size. To more fully test the potential of these SNPs in relationship with the OA, a follow-up study that explores the role of haplotypes on the genetic susceptibility to OA is desirable.

## 5. Conclusion

In summary, we have identified that the rs1871054 variant within the ADAM12 gene is a risk factor for increased osteoarthritis susceptibility and severity.

## Figures and Tables

**Table 1:** The summary of the basic characteristics of the groups.

Clinical characteristics		Controls	OA patients	*P* value
Number		200	164	
Age (years)		65.9 ± 5.3	67.4 ± 4.2	n.s
Male/female		62/138	58/106	n.s
BMI (kg/m^2^)		24.4 ± 4.5	25.3 ± 4.2	n.s

KL grade	1	/	0
	2	/	73
	3	/	58
	4	/	33

**Table 2:** The comparison of the 4 SNPs between the cases and controls.

Group	Genotype (frequency) rs1278279							
	G (%)	A (%)	GG	GA + AA	GG + GA	AA	GG	AA
Control	76.5	23.5	121	79	185	15	121	15
Case	75.0	25.0	92	72	154	10	92	10
OR^a^	/	1.06	/	1.17	/	0.83	/	0.88
95% CI^a^		0.87 to 1.34	/	0.79 to 1.81	/	0.35 to 1.84	/	0.36 to 2.03
*P* ^a^	/	n.s	/	n.s	/	n.s	/	n.s

Group	Genotype (frequency) rs3740199							
	G (%)	C (%)	GG	GC + CC	GG + GC	CC	GG	CC

Control	51.0	49.0	51	149	153	47	51	47
Case	52.4	47.6	44	120	128	36	44	36
OR^a^	/	0.93	/	0.92	/	0.90	/	0.87
95% CI^a^		0.76 to 1.14	/	0.58 to 1.49	/	0.54 to 1.52	/	0.48 to 1.61
*P* ^a^	/	n.s	/	n.s	/	n.s	/	n.s

Group	Genotype (frequency) rs1871054							
	T (%)	C (%)	TT	TC + CC	TT + TC	CC	TT	CC

Control	50.8	49.2	52	148	151	49	52	49
Case	35.7	64.3	29	135	88	76	29	76
OR^a^	/	1.84	/	1.64	/	2.65	/	2.78
95% CI^a^	/	1.57 to 2.23	/	1.01 to 2.74	/	1.72 to 4.14	/	1.56 to 4.95
*P* ^a^	/	<0.0001	/	0.0348	/	<0.0001	/	0.0012

Group	Genotype (frequency) rs1044122							
	T (%)	C (%)	TT	TC + CC	TT + TC	CC	TT	CC

Control	56.3	43.7	62	138	163	37	62	37
Case	57.9	42.1	51	113	139	25	51	25
OR^a^	/	0.94	/	0.98	/	0.76	/	0.83
95% CI^a^		0.73 to 1.12	/	0.64 to 1.57	/	0.44 to 1.39	/	0.44 to 1.53
*P* ^a^	/	n.s	/	n.s	/	n.s	/	n.s

^a^ORs and 95% CIs were estimated using logistic regression analyses and adjusted for age, gender, and BMI.
